# Bio-Based Polyurethane Foams: Feedstocks, Synthesis, and Applications

**DOI:** 10.3390/biom15050680

**Published:** 2025-05-07

**Authors:** Marta Santos, Marcos Mariz, Igor Tiago, Susana Alarico, Paula Ferreira

**Affiliations:** 1 CERES, Chemical Engineering and Renewable Resources for Sustainability, University of Coimbra, 3030-790 Coimbra, Portugal; marta.santos@uc.pt (M.S.); mmariz@eq.uc.pt (M.M.); 2 CERNAS, Research Centre for Natural Resources, Environment and Society, Polytechnic University of Coimbra, Bencanta, 3045-601 Coimbra, Portugal; 3 CFE, Centre for Functional Ecology, Department of Life Sciences, University of Coimbra, 3000-456 Coimbra, Portugal; itiago@ci.uc.pt; 4 CNC-UC, Center for Neuroscience and Cell Biology and CIBB, Center for Innovative Biomedicine and Biotechnology, University of Coimbra, 3004-504 Coimbra, Portugal; salarico@cnc.uc.pt; 5 Applied Research Institute, Polytechnic University of Coimbra, Rua da Misericórdia, Lagar dos Cortiços—S. Martinho do Bispo, 3045-093 Coimbra, Portugal

**Keywords:** bio-based polyurethane foams, bio-polyols, renewable sources

## Abstract

Polyurethanes (PUs) are extremely versatile materials used across different industries. Traditionally, they are synthesized by reacting polyols and isocyanates, both of which are petroleum-derived reagents. In response to the demand for more eco-friendly materials, research has increasingly focused on developing new routes for PU synthesis using renewable feedstocks. While substituting isocyanates remains a greater challenge, replacing fossil-based polyols with bio-based alternatives is now a promising strategy. This review explores the main natural sources and their transformations into bio-polyols, the incorporation of bio-fillers into PU formulations, and the production of non-isocyanate polyurethanes (NIPUs). Additionally, the study summarizes the growing body of research that has reported successful outcomes using bio-polyols in PU foams for distinct applications.

## 1. Introduction

Polyurethanes are the most consumed class of polymers worldwide, and consequently, their production has a significant economic and environmental impact [1]. In recent years, increased awareness of climate change has prompted various sectors to change their approach to reducing global pollution. This has boosted the design of new materials, prioritizing environmental considerations alongside efforts to replace conventional fossil-based materials with greener alternatives [2]. This effort has focused especially on completely or partially substituting petrol-based reagents for bio-based ones [3] in the PU field. These substitutions lead to a more sustainable production process, safer usage, proper disposal, and even an expanded range of polyurethane applications [4,5,6].

The main developments in PU synthesis have focused on using bio-polyols derived from renewable sources such as vegetable oils, lignocellulosic biomass, and carbohydrates [7,8,9]. Moreover, research has explored the incorporation of bio-fillers into PU formulations and the production of non-isocyanate polyurethanes [10,11].

In this paper, the main routes for bio-based PU production, emphasizing PU foams, are reviewed. Examples of PU foams produced using bio-polyols and bio-fillers are presented, highlighting their applications and market trends.

## 2. Background on Polyurethanes

PUs are widely used across many applications due to their complex chemical and physical properties. They account for approximately 8% of global plastic production, with 26 million metric tons produced in 2022, and are projected to exceed 31 million tons by 2030 [12].

PU synthesis relies on two main components: a diisocyanate and a polyol. In the presence of a catalyst, an exothermic polycondensation reaction between the reagents occurs, and polyurethane linkages are formed. Due to the diversity of monomers used in PU synthesis, several functional groups can be incorporated into the polymer structure, creating a wide range of materials with different characteristics and possible applications [13].

**Figure 1:**
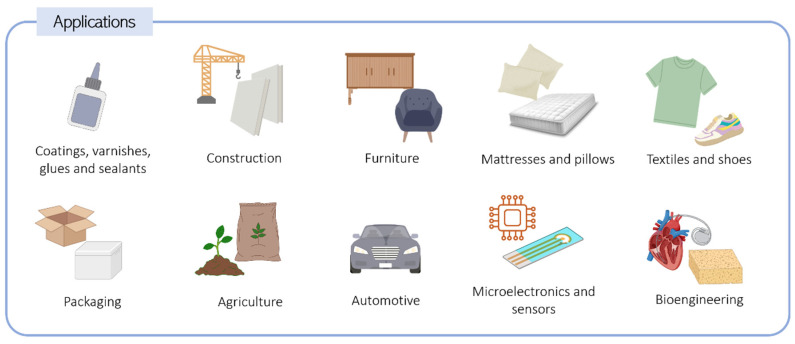
Applications of polyurethane materials across various sectors.

Based on their applicability, PU can be categorized into two main classes: flexible and rigid. Flexible PUs include coatings, sealants, fibers, adhesives, textiles, flexible foams, and elastomers, while rigid PUs comprise rigid foams, rigid polymers, wood alternatives, and composites [14]. PU foams, in particular, represent the most commonly used PU materials. Figure 1 summarizes some relevant applications of PU.

The construction sector is the most demanding of PU-based materials, and this class of polymers is used for many different purposes, such as coatings, varnishing, flooring, insulating foams, adhesives, concrete systems, road construction, and waterproofing [14,15]. Additionally, automotive, textile, footwear, and home furnishing industries extensively use PU materials [16]. Rigid PU foams are specifically prevalent in the construction and automotive sectors for use in the interior of sandwich panels [17] due to their low density, ease of processing, and high mechanical resistance. They are also applied in walls and tubes as thermal insulators as they present reduced thermal conductivity and water sorption. Refrigeration and storage sectors also consume PU foams, benefiting from these characteristics [17].

Flexible PUs have gained interest in microelectronics due to their adhesive and separation properties. Furthermore, PUs have been applied in sensor technology as membranes, owing to their strong adhesion to electrode surfaces, selectivity, and chemical stability. Thermoplastic PUs are commonly used in biomedical and tissue engineering applications as implants, scaffolds, prosthetic valves, vascular grafts, blood coagulation devices, pacemakers, and catheters because of their exceptional mechanical properties, biodegradability, hemocompatibility, and biocompatibility [18,19,20,21].

## 3. Polyurethane Foams

### 3.1. Synthesis Methods of Traditional Polyurethane Foams

**Figure 2:**
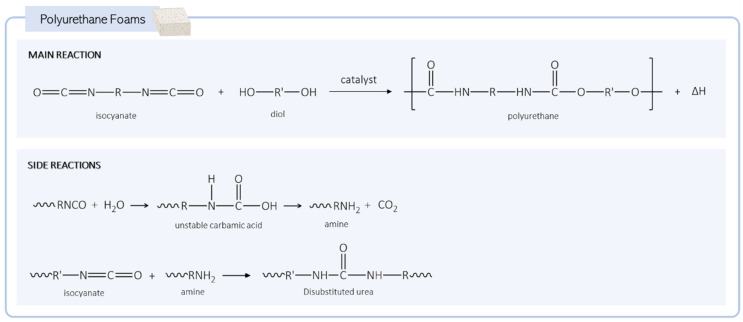
Generic reactions during the formation of polyurethane foams.

As mentioned, PUs are synthesized by the exothermic reaction of a diisocyanate with a polyol. During the synthesis, the isocyanate groups (-NCO) from the diisocyanate react with the hydroxyl groups (-OH) from the polyol and form urethane linkages (Figure 2) [22]. To obtain an expanded polyurethane (PU foam), a blowing agent must be added to the reaction mixture. There are two types of blowing agents: physical and chemical. These components are usually added to the reactional medium while the material is still in the liquid form to create the pores when the polymerization reaction occurs.

The physical agents are not involved in the chemical reaction and are usually characterized by a low boiling point that enables their evaporation when the exothermic reaction occurs. Chlorofluorocarbons (CFCs), hydrogenated fluorocarbons (HFCs), and hydrogenated chlorofluorocarbons (HCFCs) were extensively used blowing agents in the past, although, due to their negative environmental impact, their use is now limited. Other physical blowing agents, such as carbon dioxide and hydrocarbons (pentane, isopentane, cyclopentane), can also be introduced into the mixture in the gas form to expand the PU [23,24]. As for the chemical agents, adding water to the PU synthesis is the most commonly used blowing method. In this case, a side reaction of water with isocyanate occurs, producing CO_2_ and promoting the formation of pores in the matrix (Figure 2). The amine groups also formed during the reaction continue to react with isocyanate groups, forming urea groups that, at higher temperatures, can react with each other to produce biuret groups [25].

The selection of diisocyanate and polyol is crucial for defining the ultimate characteristics of the material. These two key components have a significant impact on the physical and mechanical properties of the foam. While the diisocyanate acts as the “hard” segment of the PU polymeric chains, the polyol constitutes the “soft” segment. The “hard” segment is characterized by a higher glass transition temperature (T_g_), promoting a crystalline performance and mechanical strength, whereas the “soft” segment usually exhibits a lower glass transition temperature, providing flexibility to the material. The disparity in the properties of the two components induces microphase separation within the polymeric system, leading to the diverse range of properties exhibited by PU [26]. Consequently, altering the type of reactants and their ratios enables the adjustment of the polymers’ final properties [14].

In addition to the main reactants, catalysts, chain extenders, and surfactants are common additives in PU foam synthesis and play an important role in tailoring the properties of the final material. Amines and organometallic compounds are frequently used as catalysts to accelerate the reaction, with tertiary amines being the most prevalent [27]. Chain extenders can also be added to the formulation to increase the PU foams’ molecular weight and enhance their mechanical properties, such as elasticity, tensile strength, and elongation [28]. These compounds can be aromatic or aliphatic (amine or hydroxyl-terminated with two or more functional groups) and promote the crosslinking between the “soft” segments and “hard” segments of the polymer [29]. Surfactants, while not altering the reaction, improve the cell structure by helping to stabilize gas bubbles during the foaming process. By reducing the surface tension at the pores’ interface, the surfactants prevent cell collapse and allow better control over pore size [30,31]. Silicone-based surfactants are the most commonly used in these formulations due to their excellent results in cell size uniformization and elasticity at the pore interfaces [31,32].

The selection and ratios of these additives in a PU foam formulation are crucial, as they significantly influence the material’s properties, ensuring that the performance criteria are achieved for a specific application.

### 3.2. Traditional Polyurethanes vs. Bio-Based Polyurethanes

Despite all the advantages and different uses of PU, there are also significant drawbacks associated with their production and use. The increase in global demand for PU across various sectors results in substantial waste generation during production and disposal, contributing to adverse environmental impacts [33,34]. Commonly, PU waste is discarded in landfills or incinerated. PUs are highly flammable materials that produce high smoke emissions, releasing harmful gases such as carbon monoxide and hydrogen cyanide [35]. Moreover, their production relies on fossil resources, representing both an environmental burden and an economic disadvantage, making them expensive and dependent on oil prices and availability [17].

As part of the global environmental crisis, the PU field also faces an urgent need to reduce dependence on petroleum-based materials. Over the last few years, there has been an attempt to reinvent PU and find new and more environmentally friendly production routes. Some researchers have already studied the production of PU using natural monomers as well as their biodegradability and recycling [36]. Notable progress in this domain was achieved by Quienne et al., who reported a pioneering approach that integrates the synthesis of bio-based PU foams with a closed-loop chemical recycling process, representing an important step toward the sustainable development of PU [37].

In response to the adverse impact of petroleum-based materials, there is a growing interest in developing novel PU derived from lignocellulosic biomass, vegetable oils, carbohydrates, proteins, terpenes, and rosins [14]. The underutilized potential of residues from industry and agriculture, often overlooked due to their low economic value and currently discarded, burned, or used as livestock fodder, also presents a promising opportunity [38]. Replacing fossil sources with these agricultural residues as renewable sources for bio-based materials offers a more sustainable, safe, and affordable alternative [14,39].

While traditional PUs are generally not biodegradable and persist in the environment, due to their natural origin, the bio-based alternatives tend to be more degradable. The end-of-life of PU is, therefore, also a concern. For materials built based on natural components (considered “weak chains”), waste treatment will significantly improve, as they are more easily degraded [35]. In addition, bio-based PUs can be designed to support circular economy principles through enhanced recyclability or composability, which is rarely achievable with conventional PU.

Although replacing traditional polyols with bio-polyols is the most studied approach for more sustainable alternatives, some researchers are also synthesizing non-isocyanate polyurethanes (NIPUs) that do not rely on isocyanates. However, for formulations that still depend on isocyanates, there has been growing interest in sourcing these components from renewable feedstocks. While bio-based polyols have been more studied, recent advances in bio-based isocyanates offer promising results for reducing the environmental impact. Although still less common in foam applications, a commercially available bio-based isocyanurate (STABiO™ from Mitsui Chemicals, Tokyo, Japan), as well as the recent development of fully bio-based aromatic diisocyanates from lignocellulosic raw material by Lemouzy et al., have been reported as interesting alternatives [40,41]. This aligns with recent perspectives summarized by Delavarde et al., who reviewed advancements across sustainable PU development, from bio-based monomers and NIPUs to life cycle and degradation considerations [6].

In summary, transitioning from traditional PU to bio-based PU involves not only the replacement of raw materials but also a rethinking of the life cycle of polyurethane products. Bio-based PUs present clear advantages in terms of environmental performance and resource efficiency, positioning them as key alternatives in the search for more sustainable materials.

## 4. Bio-Based Polyurethanes

### 4.1. Bio-Based Feedstocks

**Figure 3:**
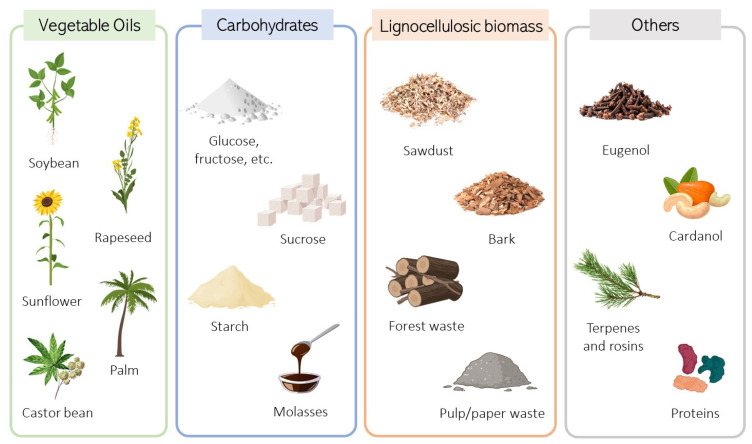
Feedstocks for renewable polyol production: vegetable oils, carbohydrates, lignocellulosic biomass, and other possible sources.

Various renewable sources have been investigated as substitutes for obtaining bio-based polyols and essential monomers to produce PU (Figure 3). Among these sources are carbohydrates, including sugars like glucose, sucrose, and starch, a common carbohydrate found in plants, and proteins from both plant and animal origins [8,35,42]. Moreover, other explored sources include vegetable oils such as sunflower, soybean, canola, palm, and castor oil, and lignocellulosic biomass (sawdust, bark, forestry droppings, and by-products from pulp/paper mills). Other plant-derived materials, such as cardanol, eugenol, and terpenes, are also being studied [43].

Vegetable oils such as castor oil, sunflower oil, soybean oil, palm oil, rapeseed oil, and Lesquerella seed oil are renewable and degradable resources that can be used in bio-polyol production. However, as the majority of these oils do not contain hydroxyl groups, some chemical modifications are required to introduce the hydroxyl groups necessary for the posterior reaction with isocyanate and urethane bond formation.

### 4.2. From Raw Materials to Bio-Polyols

Some of the raw materials must be transformed [44] to produce viable bio-polyols with hydroxyl groups accessible for further reaction. As the number of hydroxyl groups correlates with the rigidity of PU foam, when aiming for specific properties, careful consideration must be given not only to the choice of polyol but also to the modification method.

**Figure 4:**
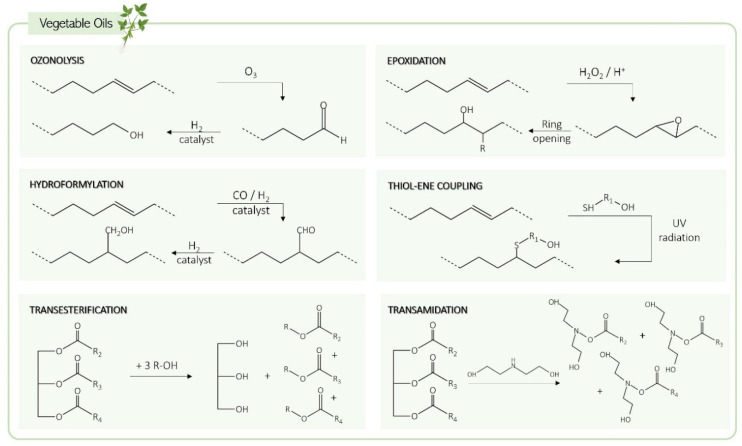
Vegetable oil transformation pathways to produce bio-polyols.

In the vegetable oils group, castor oil and Lesquerella seed oil are the only ones that contain hydroxyl groups in their basic structure, making them suitable for direct use as polyols and, consequently, more affordable and less laborious options. To use other vegetable oils in PU synthesis, their carbon–carbon double bonds or ester linkages must be modified to obtain hydroxyl groups. The main methods for this transformation include ozonolysis, epoxidation, hydroformylation, thiol-ene coupling, transesterification, and transamidation [43,45] (Figure 4).

In the ozonolysis reaction, the carbon–carbon double bond of unsaturated fatty acid chains is cleaved in the presence of ozone (O_3_), a strong oxidizing agent, resulting in the formation of an unstable ozonide that is reduced to an aldehyde by a strong reducing agent, such as sodium borohydride. Then, the aldehyde groups are further reduced to hydroxyl groups by a hydrogenation step using a Raney nickel catalyst [3]. The polyols resulting from ozonolysis can have a functionality of up to three hydroxyl groups per molecule and have low molecular weights due to the removal of the unsaturated portions of the fatty acid chains [46].

In addition to the limited conversion, due to the high reactivity of oxygen, ozonolysis can lead to side reactions and consequently undesired byproducts if it does not occur under controlled conditions [47]. It also requires high temperatures, high pressure, and toxic solvents.

The epoxidation mechanism transforms vegetable oils by using peracids (such as peroxy acid) that can be previously formed or generated in situ by reacting a carboxylic acid (acetic acid or formic acid) with hydrogen peroxide (H_2_O_2_) [48]. The epoxidation step converts the double bonds into epoxy groups [49]. Then, ring-opening is performed using ring-opening agents with hydroxyl groups, such as alcohols and inorganic acids. This step is performed at high temperatures using bases, phosphorous compounds, metals, and cationic catalysts [50].

Apart from the vegetable oil used, the type and position of the hydroxyl groups of the ring-opening agent are also very important factors for the resultant polyol and, consequently, in the final properties of the PU (they affect crosslinking, glass transition temperature, and mechanical behavior) [45]. The main drawbacks of this transformation method are the low selectivity for epoxides due to the ring opening, the use of peracids (unstable and explosive), and the corrosion promoted by the presence of strong acids [51].

Hydroformylation is the reaction that converts carbon–carbon double bonds in fatty acid chains into aldehydes through the use of a catalyst (usually rhodium or cobalt) and syngas (an H_2_ and CO 1:1 mixture) at temperatures between 70 and 130 °C [45,52]. This reaction is then followed by hydrogenation, leading to the formation of primary hydroxyl groups, which are more reactive than the secondary hydroxyl groups produced by other methods, such as epoxidation. Rhodium is the most efficient catalyst (achieving nearly 100% conversion), while cobalt (with 70% conversion) is a more economical option, emerging as the most commonly used in this type of reaction. Both compounds catalyze hydroformylation and hydrogenation reactions [46,48].

The thiol-ene coupling chemistry involves the incorporation of thiols into carbon–carbon double bonds through a single-step reaction [53]. Thiols such as 2-mercaptoethanol, 3-mercaptopropinoate, or glyceryl dimercaptoacetate are commonly used in this process, and the reaction can be promoted by using a radical initiator or a catalyst [54]. This method is highly versatile due to its tolerance for a wide range of conditions and solvents. It is also characterized as being a fast process with a high conversion rate [45]. The main factors that limit its applicability are the dependence of thiol’s reactivity on its structures and the potential side reactions with oxygen that can compromise the reaction efficiency [53,54].

Transesterification is an alternative method for obtaining polyols from vegetable oils. In this process, the ester groups of triglycerides react with polyols such as glycerol, pentaerythritol, and trimethylolpropane in the presence of a catalyst (alkaline or acid) and at high temperatures (170–200 °C) [3,45,48]. As a result, the ester bonds are converted into hydroxyl groups, and a mixture of products is obtained: mono-, di-, and triglycerides, as well as glycerol [45,55].

The transamidation procedure is similar to transesterification, except that amines are used instead of polyols. This transformation consists of the catalyzed reaction of vegetable oils with amines (usually diethanolamine, bearing hydroxyl groups) at temperatures between 100 and 120 °C [3,56].

**Figure 5:**
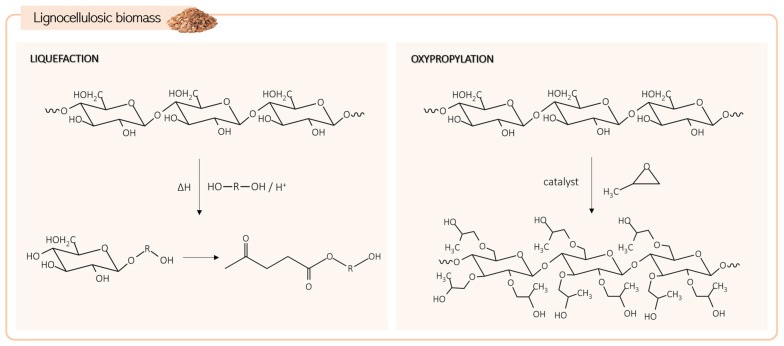
Lignocellulosic biomass transformation pathways to produce bio-polyols.

Another natural feedstock is lignocellulosic biomass, the most abundant and inexpensive renewable source. It comprises mainly three biopolymers, cellulose, hemicellulose, and lignin, in varying proportions depending on the origin. The high number of hydroxyl groups in these compounds makes them very attractive for producing bio-polyols, although their natural solid state limits their direct utilization [57]. Liquefaction and oxypropylation are the two main processes used to obtain liquid bio-polyols from lignocellulosic biomass (Figure 5) [58].

The liquefaction process involves converting solid lignocellulosic biomass into a liquid product. It is usually performed at a temperature range of 150 to 250 °C, using solvents such as polyhydric alcohols or cyclic carbonates. The reaction can be catalyzed by acids or bases such as sulfuric acid or sodium hydroxide [3,57]. During the process, solid biomass undergoes depolymerization, breaking down into smaller hydroxyl-containing molecules through the cleavage of chemical bonds within its structure [59]. Due to the amorphous nature and consequent higher susceptibility to solvents, amorphous cellulose, hemicellulose, and lignin are the first components to undergo liquefaction. With its organized structure, crystalline cellulose is less accessible, resulting in slower liquefaction (the limiting step). Solvolytic reactions decompose cellulose into glucose and other derivatives, leading to the production of compounds like levulinic acid and levulinates that form a mixture suitable for direct use in PU materials synthesis [57,58]. Optimizing liquefaction parameters such as temperature, time, solvent, catalyst, and respective ratios is crucial to mitigate concurrent recondensation reactions and increase process efficiency [58].

Considerations in the liquefaction process extend to the cost and source of solvents. Although petroleum-derived solvents are commonly used in the liquefaction process, they conflict with the purpose of producing greener polyols. Glycerol, a byproduct of biodiesel production, has emerged as a promising alternative to petroleum-based solvents, offering a more economical and environmentally friendly approach to polyol production [58].

Oxypropylation is based on grafting propylene oxide into the biomass macromolecular structure through a polymerization process in the presence of a catalyst (Figure 4). Typically, the biomass, catalyst (usually KOH), and propylene oxide are mixed directly. Alternatively, the biomass can undergo prior activation with an ethanol solution of KOH at room temperature and under a nitrogen atmosphere, followed by ethanol removal and subsequent reaction with propylene oxide. The oxypropylation reaction is commonly carried out at high pressure (650–1820 kPa) and high temperature (100–200 °C) conditions [57,60].

As previously mentioned, lignocellulosic biomass is rich in hydroxyl groups, yet these are entrapped within the molecular structure. In this modification process, the aim is not to insert hydroxyl groups but to enhance the accessibility of the OH groups by introducing longer chains derived from propylene oxide. The number of hydroxyl groups remains unchanged before and after the transformation; however, they become more available for further reaction.

### 4.3. Bio-Polyols in Polyurethane Foams

Although several types of PU can be synthesized using reactants from bio-based sources, this review paper will focus on PU foams, which are the most produced PU material worldwide.

Many researchers have reported using bio-based polyols in PU foam synthesis. Polyols from different sources have been used for partial or total substitution of standard petrol-based polyols in PU foam synthesis, showing promising results in the final material’s characteristics.

As previously mentioned, PU foams can be used in many applications where the desired characteristics can be completely distinct. Considering the final purpose of a given PU foam, the selection of the bio-polyol can change the foam behavior and/or add interesting properties to the material. Table 1 lists some of the research works reported in recent years using bio-polyols in PU foam preparation, as well as some of the main results observed.

### 4.4. Bio-Additives in Polyurethane Foams

PU foams often benefit from additives that improve their characteristics and applicability. Over the last few years, there has been increasing interest in more sustainable options, with natural fibers being the most popular bio-additives.

Fibers derived from agro-waste have been used to reinforce PU foam formulations to form composites with increased strength and biodegradability. Additionally, these composites offer advantages such as their lightweight, low density, low cost, availability, and accessibility [44].

Different vegetable or animal sources can be used to obtain natural fibers. Some examples include coconut husk, palm kernel shell, jute, kenaf, banana fiber, rice husk, wheat husk, flax, bast and cotton husk, wool, and silk [73]. However, the polar behavior of these natural fibers can jeopardize their interaction with the polymer. To tackle this, they are usually submitted to chemical or physical treatments to improve interfacial adhesion. Chemical treatments modify the fibers, improving the final composites’ adhesion, stiffness, stability, and thermal conductivity. Methods such as alkalization, salinization, acetylation, acrylation, esterification, peroxide treatment, permanganate treatment, and benzoylation treatment have been applied [74]. Physical methods modify the fibers’ surface or physical structure, also aiming to enhance the compatibility between the PU matrix and the filler. These methods do not alter the chemical composition and can include thermal and radiation treatments, stretching, and drying [44].

Biological treatments using fungi, enzymes, and bacteria are also being investigated. Fungi produce hyphae, filamentous structures that penetrate and colonize the fiber surface, creating fine holes and increasing the surface roughness [75]. Enzymes, on the other hand, are used to remove the non-lignocellulosic content from the surface of the fibers, increasing the exposure of the individual fibers and, consequently, their bonding with the PU [74,76]. Cellulose-producing bacteria can also be used to modify the fiber’s surface. These bacteria grow and produce cellulose that further attaches to the surface, altering its morphology [77].

The main disadvantage of natural fibers is their variability in properties. As most of the compounds originate from natural sources, these fibers’ characteristics depend on the growth and processing conditions [44]. Other bio-based additives such as fire retardants, colorants, and antibacterials can also be helpful in PU foam formulations. Numerous studies have reported the use of bio-fillers and modified bio-fillers to enhance PU foams’ characteristics.

Atiqah et al. studied the enhancement of physical and thermal properties of sugar palm/glass fiber-reinforced thermoplastic PU composites using different fiber treatments: 6% alkaline, 2% silane, and combined 6% alkaline–2% silane. The combined treatment resulted in composites with lower density, reduced thickness swelling and water absorption, and improved thermal stability compared to untreated materials [78].

In their study, Mohammed et al. explored the impact of potassium permanganate (KMnO_4_) treatment on sugar palm fiber-reinforced thermoplastic PU composites. Using extrusion and compression molding, they treated the fibers with KMnO_4_ following a 6% NaOH treatment. A 0.033% KMnO_4_ treatment provided the best balance of flexural strength, impact strength, and thermal stability. However, mechanical properties were lower than those treated with NaOH alone, suggesting that while KMnO_4_ enhances thermal properties, it reduces the composites’ flexural and impact strengths [79].

Glowacz et al. investigated the incorporation of sustainable biowaste fillers, specifically sunflower husks, rice husks, and buckwheat hulls, into rigid PU foams. They added these fillers in different weight ratios (5%, 10%, 15%) and observed that the modified foams exhibited higher apparent density, lower water absorption, increased compressive strength, and enhanced thermal stability [80].

In their research, Dong et al. produced a bio-based nitrogen–phosphorus flame retardant from camphene for PU foams. This additive significantly improved fire resistance and mechanical properties, increasing the limiting oxygen index by 41.7%, decreasing the maximum heat release rate by 40.3%, and increasing the compressive strength by 166%. The fire retardant promoted the formation of a phosphorus-rich char layer and released non-combustible gases during combustion, reducing the foam’s flammability [81].

Sienkiewicz et al. incorporated the natural compound curcumin into PU foams to improve their antibacterial, antiaging, mechanical, and thermal properties. Using curcumin at concentrations of 1, 2, and 5 wt %, they improved compressive and flexural strengths, reduced water uptake, and enhanced thermal stability. Foams containing 5 wt % curcumin also exhibited remarkable antibacterial activity against *Escherichia coli* (*E. coli*) and *Staphylococcus aureus* (*S. Aureus*) [82].

Zainuddin et al. explored the integration of natural dyes from turmeric powder, Kumkum powder, Telang’s flower powder, and dragon fruit peel powder into PU foams. The study focused on improving the aesthetic and mechanical properties of the materials by adding these natural pigments at 10 and 12 wt %. The results showed no change in the chemical structure of PU foams and a significant improvement in compressive strength. The vibrant colors achieved with these additives make the materials suitable for applications in household and children’s products [83].

In summary, incorporating natural fibers and other bio-based additives—such as fire retardants, colorants, and antibacterial agents—into PU foams enhances their properties, biodegradability, and cost-effectiveness, thereby expanding their range of applications.

### 4.5. Non-Isocyanate Polyurethane Foams (NIUPs)

**Figure 6:**
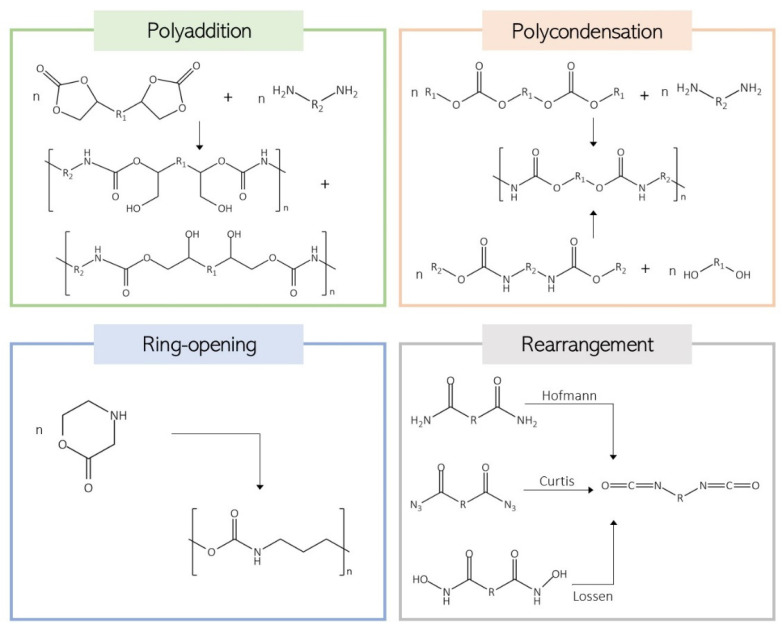
Schematic representation of the four main routes of NIUP synthesis.

Although less common, PU materials can be produced without using isocyanates. NIPU foams have emerged as a safer and more environmentally friendly alternative to avoid isocyanates [84]. These materials can be synthesized by four main approaches: polycondensation, ring-opening polymerization, rearrangement, and polyaddition (Figure 6) [15,19,85].

Polycondensation reactions can occur via two routes: between chloroformates and amines or between carbamates and alcohols, and the rearrangement reactions consist of the reaction of carboxylic derivatives undergoing rearrangement in the presence of alcohols [86] Another method is the ring-opening polymerization (ROP) of aliphatic cyclic urethanes [87]. The most commonly used route for NIPU synthesis is the polyaddition of cyclic carbonates and amines, both possessing at least two reactive moieties. This approach is isocyanate- and phosgene-free, ensuring a safer process. The nucleophilic attack of the amine on the C-C carbonyl promotes the ring opening and the formation of a carbamate linkage and a pendant hydroxyl group [88]. The PUs produced by this reaction are also referred to as polyhydroxyurethanes, as each urethane linkage generates a primary or secondary hydroxyl group. This enables the establishment of inter- and intramolecular hydrogen bonds with the urethane carbonyl groups [19,85,89]. These hydroxyl groups can react with different functional groups, enabling further functionalization, influencing the polymer properties, and improving the chemical resistance to organic solvents [89,90]. When at least one of the compounds of polyhydroxyurethanes has three or more reactive groups, the polymer can also be crosslinked. Another advantage of this route, when compared with the traditional process, is that the resultant materials are less sensitive to moisture and storage conditions and do not produce irreversible side products like urea or CO_2_ [89].

Five-membered cyclic carbonates are mainly synthesized through the conversion of epoxies using carbon dioxide (in the presence of a catalyst). They exhibit low toxicity and biodegradability, being very attractive for use as monomers in these copolymerization reactions [15,19]. These monomers can also be produced from biomass by the epoxidation of olefins and subsequent reaction of the epoxide with carbon dioxide or by transesterification of a bio-based diol with dimethyl carbonate [15]. Six- or seven-membered cyclic carbonate routes are less environmentally friendly as they require a phosgene derivative [91].

Despite the advantages offered by NIPU, some challenges remain to be addressed. These include low curing efficiency at room temperature, high viscosity, and poor solubility, which challenge polymer processing [88]. Additionally, the resultant foams often exhibit larger pore sizes than traditional ones, influencing properties such as thermal conductivity, which is essential for some applications, such as thermal insulation. Other challenges of NIPU are the higher production costs, slower foam formation, and limited mechanical properties, such as reduced flexibility [92]. However, recent works, such as that of Purwanto et al., address the significant challenges associated with the processing of NIPUs, presenting innovative strategies that increase their viability in the competitive polyurethane market. Their research highlights a rapid preparation method for reprocessable polyhydroxyurethane foams, significantly reducing synthesis time while improving mechanical properties and sustainability [84].

### 4.6. Market and Applications

A US study estimated the global market size for bio-based PU at USD 36.4 Million in 2022. The expected annual growth rate is 8% until 2030. The segments with the most significant market shares are building and construction, automotive, and consumer goods (especially packaging in the food sector and electronics). Flexible foams have been shown to be the most commonly used and most profitable PU material, accounting for 50% of the total bio-based PU consumption. These numbers are due to their wide use in mattresses, sofas, automotive interiors, and packaging applications. The Asia-Pacific region led global consumption of bio-based PU in 2022, accounting for over 38%, driven by the growth of industries such as automotive and construction in emerging economies like China and India. This market dominance is also supported by low-cost labor and accessible land, promoting the expansion of the bio-based PU market [93].

While construction and automotive remain the most promising application areas due to the demand for insulation and lightweight materials, bio-based PU foams still face regulatory and performance barriers. Current challenges include ensuring compliance with building codes (e.g., flame retardancy, thermal aging, smoke emission) and maintaining cost competitiveness. Compatibility with large-scale industrial manufacturing is also a constraint. These limitations underscore the need for further research and innovation, translating into opportunities for researchers to develop impactful work in this field and for industries to invest in these materials to meet the growing demand for more sustainable and cost-effective alternatives [73].

Studies have already shown that bio-based PU foams have the potential to replace or even surpass currently used PU foams. A study by Mohammadpour and Sadeghi demonstrated that PU foams synthesized with liquefied lignin represent a promising alternative for oil absorption applications. This alternative stands out for its bio-based origin and for exhibiting improved oil penetration and retention performance compared to existing options [63].

Członka et al. developed soybean oil-based PU foams suitable for construction and packaging applications using clove powder as a filler. The composite PU foams demonstrated resistance to compression, thermal stability, and enhanced compression, impact, and flexural strengths. Additionally, the presence of the bio-filler promoted an anti-aging effect. Furthermore, the final foams demonstrated antibacterial properties with an inhibition rate of more than 90% (*E. coli* and *S. aureus*) [94]. These antimicrobial features are particularly interesting for packaging, especially in the food and electronics sectors. Despite packaging applications offering a substantial opportunity for bio-based PU, shelf life, migration of bio-based additives, and recyclability remain critical concerns.

Considering the construction sector, Rodrigues et al. synthesized rigid PU foams using *Acacia mearnsii* tannin extract and castor oil for application as sandwich panels. Compared to fossil-based foams, the developed foams presented improved thermal and fire resistance and lower smoke and harmful gas emissions [17].

Bote et al. studied a bio-based flexible PU foam based on meso-lactide and dimer acids for car seats and headrests in the automotive industry. Characteristics such as thermal degradation, tensile and compressive properties, and tear resistance were improved with the addition of a bio-polyol [95].

In addition to structural and comfort-related uses, bio-based PU foams show potential for acoustic applications, particularly in environmental noise control. Acoustic correction of environments such as offices, studios, and industrial spaces is a growing sector for polyurethane foams. A study by Mitrevska et al. investigated the sound absorption properties of rebonded polyurethane foams. The authors demonstrated that they could effectively reduce noise across a wide range of frequencies, especially in the mid-to-high frequency ranges [96]. Other studies have shown that the open-cell structure typical of flexible PU foams can be optimized to enhance acoustic performance [97]. Recent advances have also demonstrated that incorporating natural fillers, such as bamboo leaves or rice husk particles, into bio-based PU matrices further improves their sound absorption characteristics, making them promising candidates for sustainable acoustic insulation materials [98,99]. However, structural variability in bio-based PU may limit commercial adoption unless supported by additional research data.

Bio-based PU foams can also play a significant role in the agricultural sector through applications such as the controlled release of coated fertilizers and pesticides, as well as soil stabilization for erosion prevention [100,101]. They can also be applied in irrigation systems to insulate and improve their efficiency, and in insulation for animal housing and storage facilities [100]. Despite these innovative uses of bio-based PU foams, agricultural applications remain primarily exploratory. Challenges include their environmental degradation behavior, the toxicity of their degradation byproducts, and the need for affordable large-scale production processes.

While bio-based PU foams cover a wide range of applications—including construction, automotive, packaging, acoustic insulation, agriculture, and biomedical uses—each of these sectors presents distinct challenges and maturity levels. Construction and automotive applications currently lead commercialization due to well-established processing infrastructure and the demand for thermal insulation. However, mechanical strength, flammability, and long-term durability limitations can still limit the widespread substitution of fossil-based PU in structural roles. In packaging and consumer goods, the low cost of fossil-based alternatives remains a competitive challenge despite the sustainability appeal of bio-based options. Acoustic insulation appears especially promising due to the natural open-cell structure of flexible foams, yet more standardized testing and material consistency are required for regulatory acceptance. Agricultural uses, on the other hand, are primarily at a proof-of-concept stage and must overcome challenges related to safety and environmental performance under real-life conditions. These varying degrees of maturity underscore the need for specific formulations, lifecycle assessments, and pilot-scale validation to bring bio-based PU foams closer to market viability.

### 4.7. Biomedical Applications

Medical and healthcare products can also have bio-based PU in their composition. Wound dressings, scaffolds, and implants such as plates, pins, screws, and rods can benefit from the biocompatibility of these polymers. In Europe and North America, an increase in the use of bio-based PU in the medical industry is expected [93].

PU foams are particularly useful as wound dressings. Due to their porous nature, they promote gas and moisture exchange and present low adhesion to the wound area, minimizing skin trauma during dressing removal [5,102]. Additionally, they can be used as scaffolds for tissue engineering due to their three-dimensional network, where cells can proliferate and differentiate [7,103].

A biodegradable lignin-based PU foam was studied by Li et al. for application as a wound dressing. Through a green synthesis method, the authors synthesized an NIPU foam with incorporated silver nanoparticles to provide antimicrobial properties. The resulting composite exhibited excellent mechanical and thermal stability and more than 95% antibacterial efficacy against *E. coli* and *S. aureus*, promoting rapid wound healing in vitro [104].

Another study involving the incorporation of silver during the synthesis of lignin-based PU foams was conducted by Li et al. The phenolic hydroxyl groups of liquefied lignin act as a direct reducing agent and capping agent for silver ions, forming silver nanoparticles in the PU matrix. The final material showed good mechanical and thermal properties, biocompatibility, and biodegradability. The foams also presented an antibacterial rate of more than 99% against *E. coli* and *S. aureus* within 4 h. With these characteristics and the ability to absorb exudates and promote wound healing of the full-thickness defects, these lignin-based PU foams were considered suitable for use as wound dressings [105].

Also, for wound dressing applications, Bužarovska et al. synthesized a composite of fatty-acid-based PU foam, polylactic acid (PLA), and zinc oxide nanoparticles using the thermally induced phase separation method. The composite exhibited high porosity, favorable interactions with fibroblast cells, and no toxicity. The materials showed suitable water uptake, hydrophilic properties, and effective antibacterial activity against *S. aureus* and *Pseudomonas aeruginosa* [106].

Bahatibieke et al. developed an injectable, in situ foaming porous PU scaffold containing castor oil and gelatin for cartilage regeneration. The scaffold adapts to irregular cartilage defects, allowing a dynamic microstructure that retains substances and promotes cartilage repair. Compared to traditional scaffolds, the material demonstrated superior repair capabilities, minimal cytotoxicity, and effective chondrogenic differentiation [107].

A peptide-based PU foam was synthesized by Zhang et al. and showed promising results to be used as a scaffold for the supported growth and phenotype of bone marrow stromal cells (BMSC) for 30 days. The material was produced using lysine-di-isocyanate and glycerol, decomposed into non-toxic components, and did not significantly alter the solution’s pH, ensuring excellent biocompatibility. The material also presented good thermal stability in water, allowing autoclave sterilization and possessing physical properties suitable for tissue engineering applications [108].

Despite their promising functional properties, bio-based PU foams face significant challenges in biomedical applications. Relevant issues include the reproducibility of bio-polyol compositions, in vivo degradation behavior, and compatibility with sterilization processes. Moreover, while lab-scale results are promising, the shortage of industrial-scale production routes tailored for medical-grade foams limits their commercial viability. Additionally, regulatory approval processes (e.g., FDA or EMA certification) are time-consuming and costly, further delaying clinical implementation. For these reasons, current biomedical applications remain at pre-clinical or experimental stages, with translation to market still under development.

## 5. Concluding Remarks

This review has highlighted the main developments in producing bio-polyols for the PU industry. The performance of these PU materials is promising, and many possibilities arise for substituting fossil-based PU with more sustainable options.

Vegetable oils, due to their abundance and versatility, have emerged as excellent raw materials for the production of quality bio-based PU. However, their use must be balanced against competition in the food industry, which raises ethical considerations [100]. Carbohydrates derived from resources such as corn and potatoes can also affect the food supply chain.

Other alternative sources, such as lignocellulosic biomass derived from non-edible crops or forest waste, are also being studied and offer advantages in sustainability and non-competition with food resources. However, many technologies needed to process these complex materials remain economically unviable for industrial production.

As for the NIPUs, although they are entirely fossil-free and advantageous from an environmental point of view, they are not yet ready for large-scale production due to processability and economic challenges.

The implementation of circular economy principles, such as recycling PU and using waste products, are the most promising paths to further reduce both the costs and the carbon footprint associated with PU manufacturing [109].

In conclusion, there are many possibilities for advancing toward more eco-friendly PU production. The transition from lab-scale research to industrialization is essential for the widespread implementation of these materials. With the intensification of the emphasis on sustainability, more and more research has been dedicated to studying these materials, and more industries are open to using bio-based reagents in their product formulations. A strong collaboration between academia and industry can be the key to developing ethical and economically viable solutions that do not significantly compromise the cost-effectiveness of the final products [2].

## Figures and Tables

**Table 1:** Recent research on bio-based polyols used in PU foam synthesis: polyol source, formulation, and resulting properties.

Polyol Source	Formulation	Results	Refs.
Sugar beet pulp-based polyol (SBpol)	**Polyol:** up to 100 parts per hundred (php) of SBpol replacing petroleum-based polyol (GL-400) SBpol synthesized using an acid-catalyzed solvothermal liquefaction **Isocyanate:** 141–186 php methylene-4,4′-diphenyl diisocyanate (pMDI) **Blowing catalyst:** 0.7 php Tetramethylenediamine **Gelling catalyst:** 0.2 php dibutyltin dilaurate (DBTDL) **Surfactant:** 2 php TEGOSTAB^®^ B 8467 **Blowing agent:** 3 php water	Decreased close-cell content and increased water sorption, increasing the amount of SBpol Superior insulation and mechanical properties when compared to petroleum-based foams Thermal stability up to 247 °C Unsatisfactory flame-retardant properties	[39]
Liquefied sugar-cane bagasse	**Polyol:** polyethylene glycol with 10 to 50% replacement with bio-polyol **Isocyanate:** 1.1–1.5 isocyanate index; methylene diphenyl diisocyanate (MDI) **Catalyst:** 0.1–0.5 php N,N-dicyclohexylamine **Surfactant:** 0.5–2.5 php silicon surfactant **Blowing agent:** 0.5–2.5 php water	Increased density and decreased compressive strength with higher bio-polyol content Heterogeneous surface and irregular pore shape with 30% bio-polyol in the polyol mixture	[61]
Sucrose	**Polyol:** 15 wt % sucrose **Isocyanate:** up to 85 wt % toluene diisocyanate (TDI) **Catalyst:** 2-ethylhexanoate (Tin (II)) **Additives:** up to 80 wt % propolis **Blowing agent:** water	Enhanced antibacterial activity (92.7% reduction with 20 wt % propolis) PU foam derived from sucrose and propolis undergoes degradation through an environmentally friendly process	[33]
Sucrose	**Polyol:** 10–35 wt % sucrose **Isocyanate:** 65–90 wt % poly-HDI **Catalyst:** Tin (II) **Blowing agent:** water	Uniform cellular structure Apparent density (0.114–0.126 g/mL) and thermal conductivity (0.049–0.053 W/(m·K)) within the traditional foam range Best results for 15 wt % sucrose and 85 wt % poly-HDI Enzymatically degradable. The decomposition products can be used to resynthesize PU foams	[36]
Palm oil	**Polyol:** polyether polyol mixture (PPG5100 and PPG750, 7:3) and palm oil polyol with 0–45% of bio-polyol. **Isocyanate:** Polymethylene polyphenylene isocyanate **Catalysts:** ZF-10 and LE310 **Crosslinking agent:** triethanolamine (TEOA) **Surfactant:** 8920LO **Blowing agent:** water	Decreased open cell content and cell size Decreased swelling ratio. Increased T_g_ and compressive strength. Thermal stability not significantly affected. Optimal percentage of polyol dependent on the intended application	[62]
Bio-oil from sugarcane bagasse	**Polyol:** 50 wt % polyol (up to 100 wt % substitution of polypropylene glycol with bio-oil produced by hydrothermal liquefaction) **Isocyanate:** 43.2 wt % pMDI **Catalysts:** 2.3 wt % DBTDL and 2.3 wt % DABCO 33lV **Blowing agent:** 2.2 wt % water	High amounts of bio-oil improved thermal stability in 350–450 °C range Less bio-oil (<50%) results in more compact pores Excellent compressive strength (up to 450 kPa))	[34]
Liquefied lignin	**Polyol:** 50 wt % polyol (up to 100 wt % substitution of polypropylene glycol triol with bio-oil produced by microwave liquefaction) **Isocyanate:** 43.2 wt % pMDI **Catalysts:** 2.3 wt % DBTDL and 2.3 wt % DABCO 33lV **Blowing agent:** 2.2 wt % water	Increased lignin polyol content leads to a more disorganized structure and lower density Enhanced heat stability, glass transition temperature, and compressive properties Improved oil adsorption properties.	[63]
Oil from fruit seeds	**Polyol:** 32.9 wt % polyol (with 75% of petrochemical polyol replaced by bio-polyols from different seed oils, obtained by transesterification) **Isocyanate:** 65.1 wt % 4,4′-diphenylmethane diisocyanate **Surfactant:** 0.7 wt % Niax Silicone SR-321 **Blowing agent:** 1.3 wt % water	Elimination of the catalyst due to triethanolamine incorporation in the bio-polyols Highest closed cell content (45% vol.) with pomegranate-seed-based polyol Good thermal insulation (32–35 kW/m·K) Density of approximately 40 kg/m^3^ Good dimensional stability and compressive strength (100–250 kPa)	[64]
Mixture of bio-polyols	**Polyol:** Petrochemical polyol replaced by a mixture of bio-polyols (20–40 php) obtained by epoxidation and opening of oxirane rings **Isocyanate:** pMDI **Catalyst:** Polycat 218 **Surfactant:** Niax silicone L-6915 **Blowing agent:** water	Dimensionally stable at room temperature Thermal conductivity comparable to reference foams Enhanced insulation properties due to increased cellular density Slight reduction in compressive strength but acceptable but with acceptable mechanical properties when compared to reference ones	[65]
Rapeseed oil	**Polyol:** Up to 30 wt % of bio-polyols derived from rapeseed oil with different hydroxyl values to replace petrochemical polyol **Isocyanate:** pMDI **Filler:** 0.5–2 wt % of microcellulose **Blowing agent:** water	Tensile strength between 156–264 kPa Elongation at break of 310–510% Hardness of 8.1–23.1 kPa Comfort factor between 3.1–7.1. Decreased sound insulation when compared with reference foams	[66]
Soybean, Linseed oils	**Polyol:** 0–44 pphp (parts per hundred parts) substitution of polypropylene polyol by bio-polyols obtained by epoxidation 10.6–13 pphp Glycerol **Isocyanate:** 62–99 pphp TDI **Catalysts:** 0.1 pphp dibutyltin dilaurate (DBT) and 0.6–0.7 pphp DBACO **Surfactant:** 3.8–4.4 pphp silicone surfactant **Blowing agent:** 2.6–5.3 pphp water	Apparent density of 40–90 kg/m^3^ Open-cell structure; uniform shape and size Improved Young’s modulus and compression deflection compared to reference foams	[67]
Used rapeseed cooking oil	**Polyol:** 23.4 wt % polyol obtained by epoxidation of the used oil **Isocyanate:** 63.9 wt % pMDI **Foaming catalyst:** 1.2 wt % Polycat^®^37 **Gelling catalyst:** 0.5 wt % KOSMOS^®^19 **Surfactant:** 0.4 wt % TEGOSTAB^®^B 8870 **Cell opener:** 0.1 wt % ORTEGOL^®^500 **Flame retardant:** 7.0 wt % TEP **Blowing agent:** 3.5 wt % water	Apparent density 12.4–13.3 kg/m^3^ Thermal conductivity coefficients 36.6–38.2 mW/m·K Closed cell content < 10% Best results with bio-polyols having epoxy values of 0.21–0.22 mol/100 g Comparable to commercial products	[68]
Olive oil	**Polyol:** replacement of ARCOL Polyol 1374 with up to 35 wt % of bio-polyol (obtained by epoxidation and ring-opening) **Isocyanate:** 51.27–65.68 wt % MDI **Blowing catalyst:** 0.1 parts by weight (pbw) DABCO NE 300 **Gelling catalyst:** 0.28 pbw DBTL-DABCO T-12 **Surfactant:** 0.15 pbw TEGOSTAB^®^ B8773 LF2 **Blowing agent:** 3.7–3.9 wt % water	Superior structural, morphological, mechanical, and thermal properties using a mixture of polyols than using only bio-polyol Higher performance than fully fossil-based foams	[69]
Microalgae oil	**Polyol:** Daltolac R570 fossil polyol replaced by 25–75 wt % bio-based polyol (epoxidation and ring opening) **Isocyanate:** pMDI with an NCO/OH molar ratio of 1.15 **Blowing catalyst:** N,N-Dimethylcyclohexylamine (DMCHA) **Surfactant:** 2.5 pbw TEGOSTAB^®^ B84501 **Flame retardant:** 10 pbw tris(1-chloro-2propyl) phosphate (TCPP) **Blowing agent:** Isopentene and demineralized water (1.6 pbw)	Low thermal conductivity (24.0 mW/(K·m)) with 25 wt % bio-polyol Slightly smaller cells compared to fossil-based foams Compression strength of 290 kPa A similar density to the reference one obtained for a catalyst-free foam	[70]
Yaupon holly powder	**Polyol:** 15.1 wt % microwave liquefaction polyol and 15.1 wt % glycerol-based polyol **Isocyanate:** 66.2 wt % pMDI **Catalyst:** 1.2 wt % DMACHA **Surfactant:** 1.2 wt % Dow Corning 193 **Blowing agent:** 1.2 wt % water	Decreased solid residue with higher liquefaction temperature (120–200 °C) and slightly increased when temperature increased to 200 °C Lower hydroxyl number at higher temperatures, decreasing urethane bond density. Better thermal stability than fossil-based foams Enhanced thermal stability with solid residue presence	[71]
Coffee grounds waster	**Polyol:** acid liquefaction of coffee grounds **Isocyanate:** MDI **Catalyst:** Polycat 34 **Surfactant:** TEGOSTAB^®^ B8404 and Dabco DC3043 **Blowing agent:** Dichloromethane	Good viscoelastic behavior Excellent geometry recovery after 70% compression Thermal stability suitable for thermal insulation Promising for acoustic insulation and aeronautics	[72]
Cellulose	**Polyol:** 10 g polyol obtained from cellulose through hydroxyalkylation **Isocyanate:** pMDI with 1.0–1.2 index **Catalyst:** 0.03–0.25 g TEA **Surfactant:** 0.31 g Silicon L-6900 **Blowing agent:** 2–3 wt % Water	Properties similar to conventional rigid foams Improved thermal stability (resistant to long-term heating at 150 °C). Thermal exposure with improvement in compressive strength Good biodegradability (70–80% degradation in 28 days at lab scale)	[9]

The examples presented in Table 1 demonstrate the significant progress made toward using bio-based polyols for PU foam synthesis. Incorporating these bio-polyols has the potential to equal or enhance the material’s properties while simultaneously decreasing dependence on petroleum-derived resources.
